# G-quadruplex DNA and RNA in cellular senescence

**DOI:** 10.3389/fragi.2024.1491389

**Published:** 2024-10-09

**Authors:** Rocio Diaz Escarcega, Paul Marshall, Andrey S. Tsvetkov

**Affiliations:** ^1^ Department of Neurology, University of Texas McGovern Medical School, Houston, TX, United States; ^2^ College of Health and Medicine, The Australian National University, Canberra, ACT, Australia; ^3^ The University of Texas Graduate School of Biomedical Sciences, Houston, TX, United States; ^4^ UTHealth Consortium on Aging, The University of Texas McGovern Medical School, Houston, TX, United States

**Keywords:** G-quadruplex, senescence, aging, age-associated disease, DNA and RNA

## Abstract

Normal cells divide, are damaged, and are repaired across their lifetime. As cells age, they enter cellular senescence, characterized by a permanent state of cell-cycle arrest triggered by various stressors. The molecular mechanisms that regulate senescent phenotypes have been actively investigated over the last several decades; however, one area that has been neglected is how G-quadruplex (G4) DNA and RNA (G4-DNA and G4-RNA) mediate senescence. These non-canonical four-stranded DNA and RNA structures regulate most normative DNA and RNA-dependent processes, such as transcription, replication, and translation, as well as pathogenic mechanisms, including genomic instability and abnormal stress granule function. This review also highlights the contribution of G4s to sex differences in age-associated diseases and emphasizes potential translational approaches to target senescence and anti-aging mechanisms through G4 manipulation.

## Introduction

G-quadruplexes (G4s or G4-DNA or G4-RNA) are implicated in nearly all cellular processes, including replication, transcription, translation, and RNA metabolism (Lejault et al., 2020; Majumder et al., 2024). These non-canonical secondary nucleic acid structures are formed by guanine-rich (G) sequences. They consist of stacks of planar arrangements of four guanine bases, known as tetrads, connected by Hoogsteen hydrogen bonds, that form a flat, four-stranded secondary structure known as a G-quartet (Lejault et al., 2021). While their functions are not completely known, a newly discovered mechanism links senescent traits to abnormal stabilization of G4s (Lejault et al., 2020; Moruno-Manchon et al., 2020). However, exactly how G4 regulates senescence in aging and age-related diseases at a molecular level remains poorly understood.

Cellular senescence prevents the division of old or damaged cells. Senescent cells undergo irreversible cell-cycle arrest, showing prominent morphological alterations accompanied by a shift in metabolism, modifications in their epigenetic landscapes, and altered autophagy, among other changes (Salama et al., 2014; Roger et al., 2021). Cells enter senescence due to many factors, both intrinsic and extrinsic, such as disruptions in proteostasis, mitochondrial impairment, inflammatory stimuli, and nutrient deprivation (Di Micco et al., 2006; Kuilman et al., 2010; Passos et al., 2010; Pazolli et al., 2012; García-Prat et al., 2016; Mikuła-Pietrasik et al., 2020). A hallmark of senescent cells is an inflammatory state characterized by a senescence-associated secretory phenotype (SASP) (Wang et al., 2024). Senescent cells also display senescence-associated β-galactosidase activity due to increased lysosomal activity (Martínez-Zamudio et al., 2021). Regions of the chromatin, including telomeres, centromeres, and retrotransposons, undergo changes in organization during senescence (Wang et al., 2024; Criscione et al., 2016).

In this review, we describe the roles of G4s in modulating the chromatin structure and the epigenetic processes that contribute to cellular senescence and how they may contribute to the open question of G4’s role in disease.

## G4-DNA or G4-RNA

**FIGURE 1:**
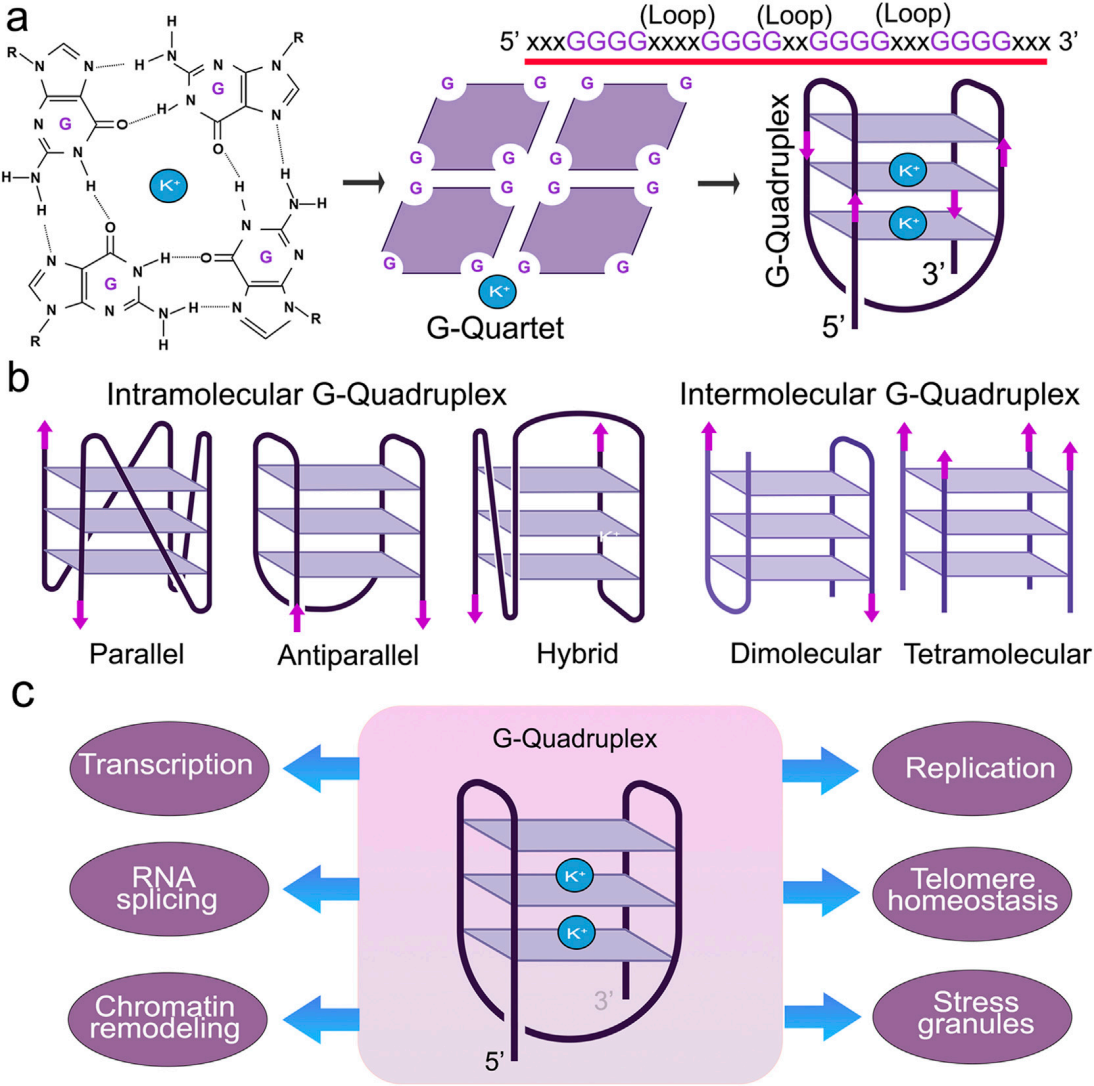
Structure and function of G4s. **(A)** Guanine-rich nucleic acid sequences are held by Hoogsteen base-pairing to form a highly stable G-quartet structure. The metal ion K+ stabilizes the stacked tetrads. **(B)** G-quadruplexes form different confirmations. **(C)** G4 structures regulate many molecular and cellular functions.

**FIGURE 2:**
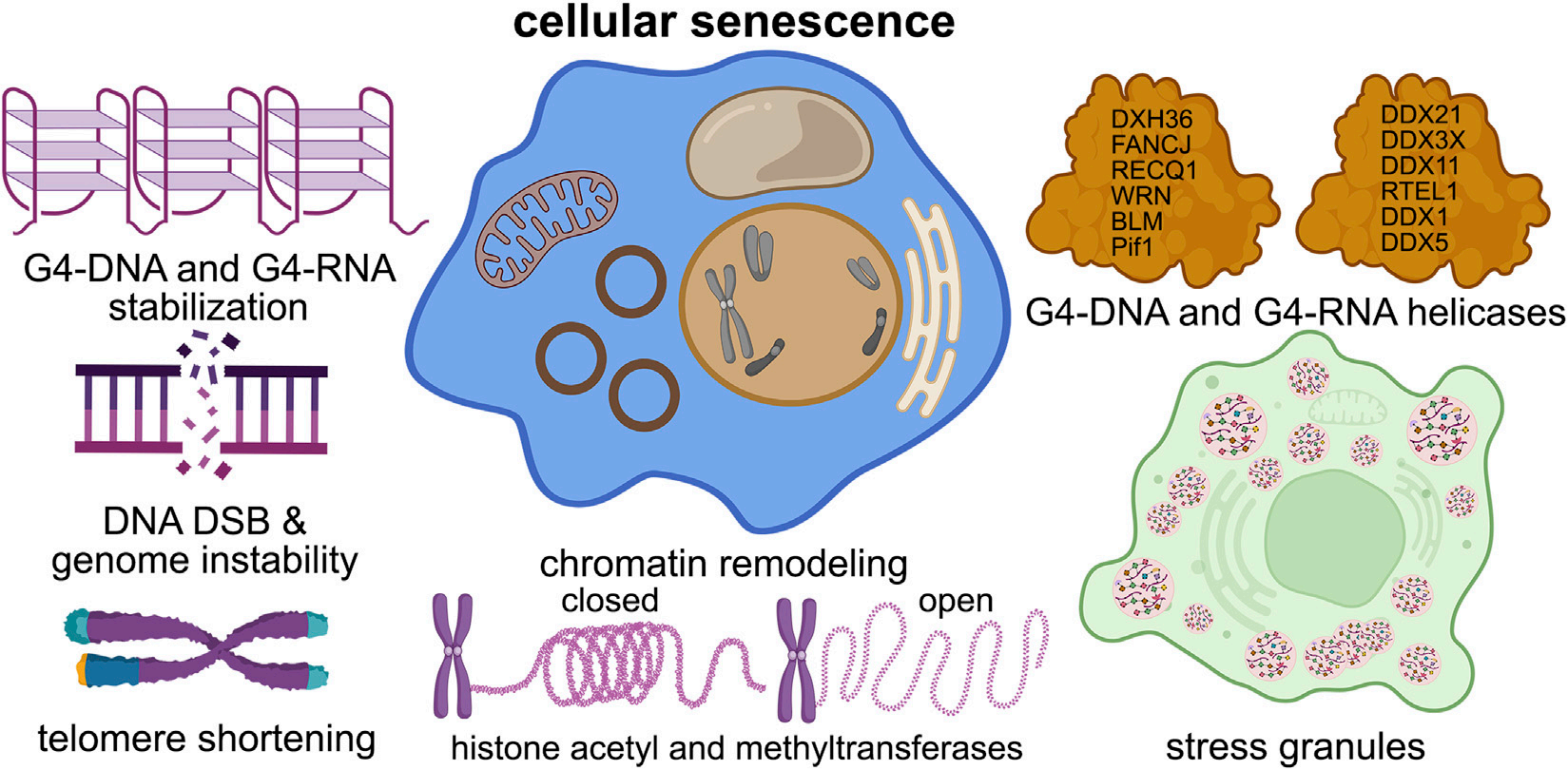
G4s contribute to cellular senescence. G4 structures contribute to nearly all of the senescence pathways in the cell, such as DNA damage, telomere shortening, chromatin structure, stress granule formation, and helicase dysfunction.

G4s are stabilized by monovalent cations, such as potassium (K^+^) or sodium (Na^+^), where K^+^ is much more effective than Na^+^ (Lopina et al., 2024; Luo et al., 2023). G4s display notable polymorphisms that allow them to assume various structures influenced by factors, such as the number and orientation of strands and the length and composition of loops (Ma et al., 2020) (Figure 1). G4 structures exhibit a spectrum of conformational states, such as intramolecular, intermolecular, and atypical configurations (Lipps and Rhodes, 2009; Varshney et al., 2020). Intramolecular structures demonstrate diverse topologies, including parallel, antiparallel, and hybrid arrangements. Yet, the physiological conditions *in vivo* tend to favor a parallel G4-DNA conformation (Lejault et al., 2021; Kan et al., 2007; Xue et al., 2007). G4-DNA structures readily form in gene promoters, telomeres, DNA replication origins, immunoglobulin heavy chain gene switch regions, mitochondrial DNA, and nucleosome-depleted regions (Besnard et al., 2012; Damas et al., 2012; Marsico et al., 2019; Tang and MacCarthy, 2021; Rawal et al., 2006; Huppert and Balasubramanian, 2007; Blackburn, 1991). G4s are implicated in nearly all cellular processes, including replication, transcription, translation, and RNA metabolism (Lejault et al., 2020; Majumder et al., 2024) (Figure 2). G4-seq analysis revealed more than 700,000 G4-DNA-forming sequences within the human genome, highlighting their significance in various biological processes (Huppert and Balasubramanian, 2005; Maizels and Gray, 2013). Interestingly, in aging cells, overly stabilized G4-DNA results in enhanced DNA damage, making these intricate structures a compelling focus for research into cellular senescence (Tabor et al., 2021; Moruno-Manchon et al., 2017). For example, upon UV irradiation, G4-DNA structures accumulate in the cell nuclei, leading to the recruitment of the Zuotin related factor 1 (ZRF1) to G4s that ensure genomic stability (De Magis et al., 2023). The absence of ZRF1 triggers the accumulation of G4 and entry into senescence (De Magis et al., 2023).

As evidenced by *in vitro* experiments (Lyu et al., 2021), G4-RNA has greater thermal stability than G4-DNA. G4-RNA forms within the untranslated regions at the 5′ and 3′ ends of mRNA and in non-coding RNA. Like G4-DNA, these RNA structures influence numerous facets of RNA functionality, including mRNA transport, maturation, degradation, splicing, miRNA regulation, PIWI-interacting RNA generation, stress response, and the reorganization of ribosomal RNA (Darnell et al., 2001; Marcel et al., 2010) (Figure 2). Understanding how G4-RNA regulates senescence is unknown.

## Helicases in cellular senescence

One set of proteins called G4 helicases may give insights into how G4-DNA and RNA are involved in the senescent state(s) (Figure 2). Helicases are ATP (or GTP)-fuelled enzymes involved in nearly all processes of nucleic acid function, such as DNA replication and recombination, RNA transcription and translation, DNA repair, telomere maintenance, ribosome biogenesis, pre-mRNA splicing, and stress granule regulation (Lohman and Bjornson, 1996; Jankowsky et al., 2011; Juranek and Paeschke, 2022; Sauer and Paeschke, 2017). Helicases can unwind standard double-stranded DNA, DNA-RNA, and complex structures (e.g., Holliday junctions, double Holliday junctions, and G4-DNA and/or G4-RNA) (Paeschke et al., 2010). Unwinding of the G4 structures can result in replication stress and genomic instability (Teng et al., 2021) (Figure 2).

Many G4 helicases are linked to human diseases of aging (Brosh and Matson, 2020; Lerner and Sale, 2019; Antcliff et al., 2021). Helicase WRN is mutated in Werner syndrome, which is characterized by accelerated aging, cardiovascular disease, and cancer (Lerner and Sale, 2019; Epstein et al., 1966). Dyskeratosis congenita, characterized by severe multisystem and bone marrow failure, is linked to mutations in RTEL1, a helicase that processes telomeric G4-DNA (Lerner and Sale, 2019; Walne et al., 2013). In Fanconi anemia, the FANCJ G4 resolving helicase is mutated and leads to cancer (Lerner and Sale, 2019; Wu and Brosh, 2009). Mutations in the helicase XPD lead to xeroderma pigmentosum and Cockayne syndrome (Lerner and Sale, 2019). Mutations in the telomere maintenance complex (the CST complex; CTC1, STN1, and TEN1) result in severe multisystem Coats plus syndrome (Simon et al., 2016). Mutated helicase BLM causes Bloom syndrome, which is associated with cancer (Lerner and Sale, 2019). Mutations in RECQ4 are associated with Rothmund-Thomson syndrome, Baller-Gerold syndrome, and RAPADILINO syndrome, which are characterized by premature aging (Lu et al., 2017). Senescence induced by RECQL4 dysfunction contributes to Rothmund–Thomson syndrome features in mice (Lu et al., 2014). The L319P mutation in the helicase PIF1 increases the risk of cancer (Chisholm et al., 2012). All these diseases, except for PIF1L319P-linked cancer, are characterized by brain pathology and aging phenotypes. Yet, how helicases affect senescence is still poorly understood.

DEAH-box helicase 36 (DHX36) is ubiquitously expressed in humans and mice. DHX36 knockout is embryonically lethal, showing that DHX36 is vitally important (Lai et al., 2012). DHX36 conditional knockdown in the hematopoietic system causes hemolytic anemia, reduced proliferation and cell-cycle defects, ineffective differentiation of progenitors and stem cells, and deregulation of genes containing a G4 DNA motif in their promoters (Lai et al., 2012). Intriguingly, G4-DNA is required to transiently silence and activate genes critically involved in learning and memory in mice (Marshall et al., 2024). Site-specific resolution of G4-DNA by dCas9-mediated deposition of the helicase DHX36 impairs fear extinction memory, possibly implicating DHX36 in age-associated cognitive impairment (Marshall et al., 2024). As DNA repair and maintenance mechanisms decline with age, DHX36 *activity* may also diminish, leading to more stable G4s and increased genome instability (Antcliff et al., 2021; Murat et al., 2018; Chen et al., 2021). Conversely, DHX36 levels and activity could increase in senescent cells due to overly stable G4s. Helicases are crucial in resolving G-quadruplex structures and facilitating essential cellular processes, such as DNA replication, transcription, and telomere maintenance (Frobel and Hänsel-Hertsch, 2024).

## G4s in chromatin remodeling and senescence

Chromatin changes significantly with senescence, and those changes have consequences. The Balasubramanian lab reported that a significant proportion of G4s are situated within nucleosome-depleted regions and linked to active transcription, suggesting that chromatin remodeling processes determine the formation of G4-DNA (Hansel-Hertsch et al., 2016). Chromatin opening leads to a transcriptional bubble that creates a favorable environment for G4 formation. Once G4s form, they can induce further chromatin reorganization and promote epigenetic alterations, such as CpG methylation, which is important in transcription control. The stability of G4s can be influenced by CpG methylation. Depending on the topology and the position of methylated CpG sites, methylation stabilizes or destabilizes G4 (De and Michor, 2011; Lin et al., 2013; Stevens and Kennedy, 2017a; Stevens and Kennedy, 2017b).

G4-DNA motifs and DNA methyltransferases (DNMTs) interact strongly *in vitro* (Cree et al., 2016). G4s were identified within the promoter regions of the oncogene c-Myc and two imprinted genes (*MEST and CDKN1C*) (Cree et al., 2016; Hay et al., 1987; Levens, 2010). DNMT3A and DNMT3B are responsible for *de novo* methylation, and along with DNMT1, they are involved in methylation maintenance. They bind robustly to each of the three G4 structures but little or not at all to their non-G4 mutants. G4-DNA may influence establishment and perpetuation of CpG methylation and affect chromatin dynamics (Cree et al., 2016; Smith and Crocitto, 1999; Xu et al., 1999; Maloisel and Rossignol, 1998; Mao et al., 2018). To summarize, G4-DNA engages with DNMTs and transcription factors, affecting histone modifications, chromatin relaxation, and the repositioning of nucleosomes during replication and transcription.

We recently performed genome-wide gene expression analysis (RNAseq) to identify genes modulated by the G4-stabilizing small-molecule pyridostatin in primary cultured neurons. Of 18,745 genes with measured expression, 901 were differentially expressed in neurons treated with pyridostatin, indicating that G4s are an important mechanism of transcriptional regulation in neuronal cells. Intriguingly, networks of genes regulating p53 signaling, immune response, learning and memory, and cellular senescence were affected by pyridostatin (Escarcega et al., 2023). In particular, pyridostatin upregulated *Pml* and *Hmga1*, which regulate senescence (Korb and Finkbeiner, 2013; Bou Sleiman et al., 2020). The E3 ubiquitin ligase *Pirh2* (*Rchy1*), which promotes degradation of DNA damage-response proteins, was also upregulated (Escarcega et al., 2023). Our findings indicate that G4s are an important mechanism for modulating gene expression and neuronal senescence-like mechanisms, in which neurons are metabolically active but likely have information-processing deficiencies (Herdy et al., 2024).

## Histone modification and G4s

Histone modifications are associated with the regulation of cellular senescence. The changes in histones influence the structure and function of chromatin, thereby regulating gene expression and maintaining cellular homeostasis (Sun et al., 2023). Specific histone modifications influence the formation of G4 structures by altering chromatin accessibility (Sengupta et al., 2019). For example, histone acetylation, which loosens chromatin structure, might facilitate the formation of G4 structures by exposing guanine-rich sequences (Kim and Kim, 2013). Histone acetylation at specific lysine residues, such as H3K9 and H3K27, promotes G4 formation and influences genomic stability and gene expression (Sarkies et al., 2012; Komůrková et al., 2021).

In turn, G4s recruit chromatin-modifying enzymes that add or remove histone marks and influence local chromatin structure and gene expression (Reina and Cavalieri, 2020). For instance, trimethylation of H3K9me3 is associated with heterochromatin formation, where chromatin is tightly compacted and transcriptionally repressed. This compacted state generally suppresses the formation of G4 structures. In contrast, methylation at other histone residues, such as H3K4 and H3K79, sometimes correlates with increased G4 formation by altering chromatin structure and accessibility (Roy et al., 2024). This dynamic interplay between histone modifications and G4 formation is crucial in regulating gene expression and the overall epigenetic landscape. Understanding their interactions would provide valuable insights into gene regulation during senescence and the pathophysiology of age-related diseases and offer potential avenues for therapeutic intervention.

## Telomeric G4s and senescence

Treatment with G-quadruplex stabilizing ligands in human cells manifests telomeric effects, such as the depletion of telomeric proteins telomeric repeat binding factor 2 (TRF2) and protection of telomeres 1 (POT1), degradation of the telomeric guanine-rich overhang, an increase in DNA damage signals at telomeres, and impaired telomere replication (Gomez et al., 2006; Salvati et al., 2007; Rodriguez et al., 2008). Numerous G4 helicases unwind G4-DNA structures at telomeres. For example, cells lacking PIF1 exhibit DNA double-strand breaks at telomeres (Strecker et al., 2017), and the absence of BLM and WRN leads to formation of stable G4-DNA structures, particularly at telomeres (Paeschke et al., 2013; Drosopoulos et al., 2015).

Telomeres undergo transcription, yielding a long non-coding RNA (TERRA) that can form G4-RNA structures (Rudenko and Van der Ploeg, 1989). TERRA serves as a structural regulatory component of telomeres, maintaining telomere integrity and chromosomal end protection by binding to TRF2 (Chong et al., 1995). Deletion of G4-RNA structures within TERRA leads to the loss of TERRA, γH2AX-marked telomeric DNA damage, shortened telomeres, and increased telomere abnormalities (Ilicheva et al., 2015; Biffi et al., 2012; Mei et al., 2021). The DEAD-box RNA helicase DDX39 interacts with TRF2 (Yoo and Chung, 2011). Overexpression of DDX39 results in progressive telomere elongation, and its depletion leads to telomere shortening (Yoo and Chung, 2011). DHX36 binds to the telomerase RNA component, resolving G4-RNA within the telomerase RNA component and facilitating the formation of a stem-loop structure essential for telomerase reverse transcription. Stabilizing G4-RNA prevents telomere extension and leads to cellular senescence. Reducing DHX36 levels via siRNA diminishes telomerase function and shortens telomeres (Sexton and Collins, 2011; Riou et al., 2002). Clearly, telomeric G4s regulate senescence, and further exploration is necessary to delineate pathways for telomere maintenance of telomere length homeostasis, thereby averting cellular senescence.

## Stress granules, G4-RNA structures, and RNA modification

Stress granules are dynamic cytoplasmic organelles that form in response to cellular stress and help to regulate mRNA metabolism by sequestering untranslated mRNA and associated proteins. Stress granules include untranslated mRNA, translation initiation factors, small ribosomal subunits, and RNA-binding proteins (Kedersha et al., 2013; Kawane et al., 2014; Waris et al., 2014). G4-RNA is found in stress granules, where G4-RNA may assist in regulating the translation and stability of mRNA under stress conditions (Kharel et al., 2023). For example, mRNAs with 3′UTR G4-RNA structures remained more stable during starvation (Kharel et al., 2023). Furthermore, the global folding of G4-RNA induced by stress was reversed once the stress was removed. These findings suggest that G4-RNA has dynamic and potentially extensive roles in regulating mRNA metabolism as part of a stress response (Kharel et al., 2023).

The G4 helicase DDX3X binds to mRNA in stress granules, resulting in translational repression. DDX3X likely promotes the formation of stress granules under various conditions, influenced by the specific RNA-binding proteins (Marcelo et al., 2021; Zhang et al., 2024). DHX36 is also present in stress granules and helps to regulate mRNA translation and stability during stress (Chalupníková et al., 2008). The BLM helicase negatively regulates stress granule formation via unwinding G4-RNA (Danino et al., 2023). Dysregulation of helicase activity or stress granule dynamics has been implicated in various diseases, including neurodegenerative disorders (e.g., Alzheimer’s disease and frontotemporal dementia). However, the relationships among G4s, G4-helicases, stress granules, and cellular senescence are largely unexplored.

## G4 pathways and sex differences

Aging is characterized by mitochondrial dysfunction, epigenetic changes, genomic instability, dysfunctional proteostasis, including autophagy, cellular senescence, and impaired metabolism (Galkin et al., 2020; Seale et al., 2022; Labbadia and Morimoto, 2015). Many of these changes differ by sex (Hägg and Jylhävä, 2021). Sex differences are also seen in Alzheimer’s disease and related dementias, Parkinson’s disease, stroke, heart, lung, cancer, and kidney diseases, among others (Hägg and Jylhävä, 2021). Nevertheless, the hormonal environment does not fully account for the sexual dimorphism in many diseases (Hägg and Jylhävä, 2021), especially in aged individuals where circulating gonadal hormone levels are low in both sexes (Mauvais-Jarvis et al., 2020). In addition to reproductive hormones, chromosomal sex is increasingly recognized as an important factor in the sex differences in age-related diseases (Hägg and Jylhävä, 2021; Podcasy and Epperson, 2016; Honarpisheh and McCullough, 2019).

X-linked dystonia-parkinsonism (XDP) is a rare neurodegenerative disease caused by mutations in the *TAF1* (TATA-binding protein-associated factor 1) gene in the Xq13.1 region of the X chromosome (Arasaratnam et al., 2021). XDP males are affected, and females, who are heterozygous carriers, generally do not exhibit the full phenotypes (D Ignazio et al., 2022). The Richter laboratory recently showed that highly stable G4s form within mutated regions in the *TAF1* gene in patient-derived fibroblasts and neural progenitor cells (Nicoletto et al., 2024). Stable G4s reduced *TAF1* transcripts downstream and around the mutation and increased upstream transcripts. G4 destabilization with the G4 destabilizer PhpC increased the levels of the *TAF1* transcripts (Arasaratnam et al., 2021; Mitteaux et al., 2024). Proteomic analysis of striatal XDP neurons showed that neurodegenerative disease-related pathways, such as Huntington’s disease, spinocerebellar ataxia, mitochondrial function, RNA binding metabolism, and cellular senescence, were considerably represented (Tshilenge et al., 2024). Thus, the X-linked *TAF1* gene could differentially regulate senescence in males and females.

DDX3 is a member of the DEAD-box helicase family. The human and mouse genomes contain two DDX3 genes: *Ddx3x* is located on the X chromosome, and its homolog *Ddx3y* is on the Y chromosome (Kim et al., 2001; Chan et al., 2019). DDX3X is ubiquitously expressed and modulates transcription, DNA damage response, RNA splicing, and translation (Mo et al., 2021; Cargill et al., 2021; Bol et al., 2015). DDX3X elevation in cancerous cells leads to various transcriptional changes (Nozaki et al., 2014; Chen et al., 2018) and enhanced stress granule formation (Lai et al., 2013; Shih et al., 2012; Lai et al., 2022). DDX3X overexpression in adult mice leads to increased inflammation and oxidative stress (Zhou et al., 2022). Intriguingly, in HeLa cells, overexpressed DDX3X binds to and recruits the X-linked deubiquitinase ubiquitin-specific peptidase 9 to stress granules, indicating an interaction between these two X-linked proteins (Lai et al., 2022). In immune cells, DDX3X regulates the expression of p21 and IFN-β (Mo et al., 2021), likely contributing to sex differences in senescence and aging.

DDX3X has been mostly studied in cancer (Mo et al., 2021); however, recent research turned to a rare neurodevelopmental disorder—the DDX3X syndrome—characterized by intellectual disability and various and complex comorbidities, including seizures, autistic behavior, muscle weakness, and ocular and gastrointestinal abnormalities (Levy et al., 2023). Most DDX3X syndrome patients are females with *de novo* variants in DDX3X (Levy et al., 2023). Male patients have also been described with *de novo* mutations or inherited mutations from unaffected mothers (Levy et al., 2023). Monozygotic female twins with DDX3X syndrome have various clinical phenotypes due to different patterns of X chromosome inactivation (Levy et al., 2023).

DDX3Y is expressed in spermatocytes, and loss of *DDX3Y* results in a male infertility phenotype despite robust DDX3X expression in male germline cells. This observation suggests that DDX3X cannot replace DDX3Y in the male reproductive system (Foresta et al., 2000; Ramathal et al., 2015; Kotov et al., 2017; Rauschendorf et al., 2014). Notably, in mouse embryonic *Ddx3x* cKO male cortices, *Ddx3y* mRNA levels were considerably elevated, indicating a transcriptional adaptation of *Ddx3y* in response to decreased *Ddx3x* and that *Ddx3y* is expressed in neurons (Hoye et al., 2022). DDX3Y is expressed at low levels in the central nervous system (Venkataramanan et al., 2021). In cancer cells, DDX3X and DDX3Y are redundant in the context of mRNA translation (Venkataramanan et al., 2021). Yet due to a 60% difference between DDX3X and DDX3Y in their N-termini, DDX3X and DDX3Y differentially regulate translation, and stress granules contain more DDX3Y than DDX3X (Shen et al., 2022). In HeLa cells, DDX3Y promotes FUS and TDP-43 aggregation more potently than DDX3X (Shen et al., 2022). Aged cells often lose the Y-chromosome (Sano et al., 2022; Forsberg et al., 2014; Vermeulen et al., 2022), and the Y-chromosome undergoes hypermethylation in aged male cells (Lund et al., 2020). Notably, *DDX3Y* showed age-dependent methylation in blood cells (Li et al., 2022). Thus, age-dependent changes in *DDX3Y* may contribute to sex-specific senescence and aging pathways in males.

## Tools for G4 manipulation

G4s are being investigated as therapeutic targets in oncology (Zell et al., 2021). There is also a role for G4s in aging and age-associated diseases, such as Alzheimer’s disease (Vijay Kumar et al., 2023). Therefore, developing and using enhanced tools are critical. In cancer treatment, stabilizing G4-DNA leads to DNA damage and combining G4-DNA stabilizers with DNA repair inhibitors is an attractive anti-cancer strategy (Zell et al., 2021). Hundreds of G4 ligands have been synthesized, and a major challenge in the G4 field is G4 targeting, which lacks gene and cell-type specificity (Zell et al., 2021; Mendes et al., 2022; Monchaud and Teulade-Fichou, 2008). In addition, another challenge is developing therapeutics with low toxicity when killing the cell is not the goal, as it is in cancer.

Another side effect of current strategies is non-specific G4 binding. This leads to severe side effects, such as accelerated brain aging and earlier onset of age-associated brain disorders (Vijay Kumar et al., 2023). For example, injecting mice with the G4-stabilizing drug pyridostatin led to cognitive impairment and abnormally rapid brain aging in mice (Moruno-Manchon et al., 2017). Excitingly, a recent study stabilized a single G4 or several G4s of interest without affecting other G4s by combining CRISPR and G4-stabilizing ligands (Qin et al., 2024). Fusing the G4-binding protein nucleolin with a catalytically inactive Cas9 stabilized G4s in the promoter of oncogene MYC, integrin α7 (Itga7), and telomeric G4s and led to cell proliferation arrest and cell senescence (Qin et al., 2024). Thus, CRISPR-guided biotin-conjugated G4 ligands are promising for paving the way for selectivity in targeting G4s (Qin et al., 2024).

Stabilizing G4s with small-molecule ligands has also been a great tool for understanding how G4 stabilization regulates gene expression, replication, and RNA function. Yet, small molecules that destabilize or unfold G4s are only beginning to be reported. The Monchaud lab designed a G4-unfolding assay that was combined with biophysical and biochemical methods conventionally used to study interactions between G4s and their ligands. Their unique approach identified a phenylpyrrolocytosine (PhpC)-based small molecule as a prototype of G4-destabilizing small molecule (Mitteaux et al., 2021). PhpC is an efficient G4-RNA destabilizer and modulated G4-RNA landscapes in cultured cells (Mitteaux et al., 2024). It would be interesting to determine if PhpC regulates senescence pathways and if small molecules with PhpC destabilizing properties could mitigate aging and disease phenotypes in animal models. Developing additional tools with high specificity and low toxicity will be critical for the clinical use of G4-manipulating agents.

## Conclusions and future directions

G4 structures and senescence have been linked in aging cells, but much remains to be learned about the molecular mechanisms of G4 involvement in senescent pathways. Determining if senescence is modified by genetic factors, such as G4 helicases, would be particularly interesting for the aging field. The interactions of senescence pathways and G4s should be investigated as well. We are also still learning about novel G4-binding proteins that may regulate pro-senescence and anti-senescence pathways. G4 pathways have been extensively investigated in yeast and cancer cells, and expanding research efforts to other types could shed light on cell-type-specific mechanisms of cell senescence. For example, neurons and other post-mitotic cells do not divide, but they exhibit senescent-like phenotypes, and G4s likely mediate neuron-specific senescent mechanisms during neuronal aging. Except for cancer, G4s in the human context are virtually unstudied. It is not clear how mitochondrial G4s contribute to cellular senescence. Whether G4-destabilizing small molecules could act as anti-aging therapies should also be investigated. Given that there is a great interest in sex-dependent differences in aging, how reproductive hormones and chromosomal sex contribute to G4-associated cellular senescence would be of interest. Understanding the interactions between G4s and senescent phenotypes within cells may lead to identifying viable treatments for age-related diseases.
